# Selected papers from the 13th Annual Bio-Ontologies Special Interest Group Meeting

**DOI:** 10.1186/2041-1480-2-S2-I1

**Published:** 2011-05-17

**Authors:** Larisa N Soldatova, Susanna-Assunta Sansone, Susie M Stephens, Nigam H Shah

**Affiliations:** 1 Aberystwyth University, Wales, UK; 2 Oxford e-Research Center, University of Oxford, UK; 3 Jonson & Johnson Pharmaceutical Research & Development LLC, PA, USA; 4 Stanford University, CA, USA

## Abstract

Over the years, the Bio-Ontologies SIG at ISMB has provided a forum for discussion of the latest and most innovative research in the application of ontologies and more generally the organisation, presentation and dissemination of knowledge in biomedicine and the life sciences. The ten papers selected for this supplement are extended versions of the original papers presented at the 2010 SIG. The papers span a wide range of topics including practical solutions for data and knowledge integration for translational medicine, hypothesis based querying , understanding kidney and urinary pathways, mining the pharmacogenomics literature; theoretical research into the orthogonality of biomedical ontologies, the representation of diseases, the representation of research hypotheses, the combination of ontologies and natural language processing for an annotation framework, the generation of textual definitions, and the discovery of gene interaction networks.

## Introduction and background

Ontologies are a key technology for the Semantic Web and have been widely adopted across biomedicine in recent years. The adoption has largely been spurred due to challenges in data integration in biology and biomedicine that originate from a lack of data standards, agreed semantics, and common vocabularies. Ontologies offer a potential solution to this problem as they can formally define the semantics of data thereby enabling the connection of heterogeneous data sources. Ontologies are used in the development of new resources that integrate existing data and knowledge, annotate experimental data, aid information retrieval, and drive literature mining.

Bio-Ontologies has been a Special Interest Group (SIG) at ISMB for the last 13 years, providing a venue for sharing experiences and methods on the use of ontologies and their application to life sciences. Over the years, the Bio-Ontologies SIG has provided a forum for discussion of the latest and most innovative research in the application of ontologies and more generally the organisation, presentation and dissemination of knowledge in biomedicine and the life sciences. In 2010, the SIG received 27 paper submissions, 11 flash updates and 14 poster abstracts. 17 papers and 9 flash updates were selected for presentation at the meeting, out of which 10 papers have been selected for this supplement.

## Summary of selected papers

The ten papers selected for this supplement are extended versions of the original papers presented at the 2010 SIG.

The papers span a wide range of topics including practical solutions for data and knowledge integration for translational medicine [1], hypothesis based querying [3], understanding kidney and urinary pathways [7], mining the pharmacogenomics literature [10]; theoretical research into the orthogonality of biomedical ontologies [2], the representation of diseases [6], the representation of research hypotheses [3,9], the combination of ontologies and natural language processing for an annotation framework [4], the generation of textual definitions [5], and the discovery of gene interaction networks [8].

In the paper titled “The Translational Medicine Ontology and Knowledge Base: Driving personalized medicine by bridging the gap between bench and bedside” by Joanne S. Luciano, Bosse Andersson, Colin Batchelor, Olivier Bodenreider, Tim Clark, Christine K. Denney, Christopher Domarew, Thomas Gambet, Lee Harland, Anja Jentzsch, Vipul Kashyap, Peter Kos, Julia Kozlovsky, Timothy Lebo, M. Scott Marshall, James P. McCusker, Deborah L. McGuinness, Chimezie Ogbuji, Elgar Pichler, Robert L. Powers, Eric Prud’hommeaux, Matthias Samwald, Lynn Schriml, Peter J. Tonellato, Patricia L. Whetzel, Jun Zhao, Susie Stephens, Michel Dumontier, participants in the translational medicine task force of the World Wide Web Consortium’s Semantic Web Health Care and Life Sciences Interest Group (W3C HCLSIG) present the Translational Medicine Ontology (TMO) as the key component of the Translational Medicine Knowledge Base (TMKB) [1]. The TMO and TMKB focus on clinical events such as those recorded in an electronic medical record. The TMO provides a framework to integrate patient-centric data collected by different research communities, industries and practitioners, from bench to bedside. The Alzheimer’s Disease use case demonstrates the advantages of the proposed knowledge base. The project uses standard Semantic Web technologies; the ontology is represented in OWL2, the associated knowledge base implemented in RDF and queries are conducted using SPARQL. TMO is available at http://code.google.com/p/translationalmedicineontology/.

The paper “How Orthogonal are the OBO Foundry Ontologies?” by Amir Ghazvinian, Natalya F. Noy and Mark A. Musen reports the results of the investigation of how the open biomedical ontologies (OBO) follow the principle of orthogonality [2]. This principle is one of the key principles adopted by the OBO Foundry: it requires that OBO ontologies do not duplicate classes (or terms) which are already defined within other OBO ontologies. The authors analysed orthogonality of the 6 foundry ontologies, which conform sufficiently to the OBO Foundry principles, and the 53 candidate ontologies, which have commited to follow those principles. A lexical method to find correspondences between terms in different ontologies is used. The results quantify the degree of term reuse and term overlap in the OBO ontologies. The authors also analyse how orthogonality has evolved over time. The analysis produced a list of ~10,000 current overlapping terms within the open biomedical ontologies, thus facilitating progress towards orthogonality. The tool for assessing the orthogonality of ontologies is available at http://obomap.bioontology.org.

The paper “HyQue: Evaluating hypotheses using Semantic Web technologies” by Alison Callahan, Michel Dumontier and Nigam Shah presents a semantic web based system for the evaluation of user submitted hypotheses against experimental and literature-sourced evidence [3]. Hypothesis validity is checked by quering knowledge bases, inference over ontologies (for subsumption and parthood relations), and retrieval of facts stored as Bio2RDF linked data. HyQue has been tested on the evaluation of hypotheses of varying levels about galactose metabolism of *S. cerevisiae*. HyQue Knowledge Base, as the core of the system, has been constructed from manually curated *S. cerevisiae* gene network data and from SGD (*Saccharomyces* Genome Database). HyQue can evaluate typical hypotheses represented as biological events. The tool is available at http://semanticscience.org/projects/hyque

Paolo Ciccarese, Marco Ocana, Leyla Jael Garcia Castro, Sudeshna Das, and Tim Clark in their paper “An Open Annotation Ontology for Science on Web 3.0” discuss, in detail, a proposal for an Annotation Ontology (AO) for annotating scientific documents on the web [4]. AO is extensible and modular, allowing for inclusion of other formalisms. The authors aim to provide a sharable structure for dynamic integration of biomedical ontologies and the literature as it emerges. AO requirements were driven by the integration needs between biomedical web communities and biomedical text mining. The ontology has been developed in collaboration with a number of research groups, a major pharmaceutical company and a major scientific publisher. AO provides a model for document metadata that can be published and shared as open linked data. AO records associations between elements of domain ontologies represented as URIs and online scientific content, such as scientific papers, images, etc. AO is available at http://purl.org/ao/

Robert Stevens, James Malone, Sandra Williams, Richard Power and Allan Third in their paper “Automating Generation of Textual Class Definitions from OWL to English” describe a method and a prototype NLG (Natural Language Generation) method for the automatic generation of textual definitions from logical definitions and axioms [5]. The prototype has been tested on EFO (the Experimental Factor Ontology) and the developers of EFO have incorporated the generated definitions into the ontology. The fluency of the generated text has been assessed through surveys. The method for the automatic generation of definitions is effective in reducing labor intensive and time consuming production of definitions, and supports maintenance and curation of textual definitions. The NLG text definition tool can be found at http://swat.open.ac.uk/tools/

The paper “Scalable Representations of Diseases in Biomedical Ontologies” by Stefan Schulz, Kent Spackman, Andrew James, Cristian Cocos and Martin Boeker suggests a simplification of the ontological triad structure-disposition-process (SDP) suitable for the description of pathological entities [6]. The disjunctive class pathological entity represents diseases without specifying the ontological category. The proposed SDP approach has been used for the redesign of events, conditions, and episodes in SNOMED CT, where numerous diseases, processes and dispositions are ambiguous. SDP provides an immediate working solution for ontology developers who need a consistent mechanism to represent diseases.

The paper titled “Developing a Kidney and Urinary Pathway Knowledge Base” by Simon Jupp, Julie Klein, Joost Schanstra and Robert Stevens presents KUPKB (a Kidney and Urinary Pathway Knowledge Base) that integrates experimental findings with background knowledge [7]. The experimental data sets span multiple –omics data from human and animal models. The KUPKB is built using Semantic Web technologies and enables querying and inference over the data and knowledge. The KUP ontology (KUPO) describes the cells of the kidney in terms of their function and anatomical locations, and provides a schema for the data held in the knowledge base. KUPKB contains ~10,000,000 RDF triplets. KUPKB maybe accessed via http://www.e-lico.eu/kupkb.

Arzucan Özgür, Zuoshuang Xiang, Dragomir R. Radev and Yongqun He in their paper titled “Mining of vaccine-associated IFN-γ gene interaction networks using the Vaccine Ontology” show that the combination of biomedical ontologies and literature mining facilitates the discovery of gene interactions networks [8]. In the reported study, 186 vaccines defined in VO (the Vaccine Ontology) via the necessary and sufficient conditions were used to improve the literature-based retrieval of the IFN-γ and vaccine associated gene interactions. The importance of genes has been calculated with the use of four different types of centralities. Three gene networks have been discovered where the largest one includes ~1,000 nodes. The application of VO allowed the discovery of additional 38 genes and 60 interactions pertinent to IFN-γ and vaccinations. VO is available at http://www.violinet.org/vaccineontology/; and the SVM edit kernel for gene interaction extraction is available at http://www.violinet.org/ifngvonet/int_ext_svm.zip.

The paper “Representation of research hypotheses” by Larisa N. Soldatova, Andrey Rzhetsky and Ross D. King describes the representation of hypotheses as logical entities suitable for automatic processing by machines [9]. It is now likely that computers are producing the majority of hypotheses in biology, but there is still no a standardised language for recording such hypotheses. The proposed formalism for recording research hypotheses contributes to the development of such a standard. The formalism enables the representation of hypotheses in an operational form so that it is possible to design an experiment to test the hypotheses. Hypotheses can be decomposed into more specific hypotheses or generalised to more generic ones. Hypotheses are grouped into hypotheses sets and can be tested through cycles of investigations. The authors also propose a framework for automatic generation of hypotheses. The proposed formalism for hypotheses representation is based on an ontology called LABORS, which is available at http://www.aber.ac.uk/en/cs/research/cb/projects/robotscientist/results/

Adrien Coulet, Yael Garten, Michel Dumontier, Russ B. Altman, Mark A. Musen and Nigam H. Shah in their paper “Integration and publication of heterogeneous text-mined relationships on the Semantic Web” report on the PHARE (the PHArmacogenomic Relationships) ontology that is used for normalizing text-extracted relationships from the pharmacogenomics literature [10]. PHARE has been constructed semi-automatically. First, over 40,000 relations have been automatically extracted from MEDLINE abstracts, which link key entities (genes, drugs, and phenotypes) as well as modified or composite entities, such as drug effect or disease treatment. 41 genes highlighted by PharmGB, 3,007 drugs, and 4,202 phenotypes are used in the mining process to extract relationships they participate in. Secondly, a normalised set of 229 most frequent relations and 76 roles was identified by manual curation. The normalised relationships have been used to instantiate ~30,000 roles encoded as RDF triplets and are available for use on the Semantic Web: http://sparql.bioontology.org/webui/. The PHARE ontology is available at http://bioportal.bioontology.org/ontologies/45138

## Competing interests

The authors declare that they have no competing interests.
